# Diversity within the entomopathogenic fungal species *Metarhizium flavoviride* associated with agricultural crops in Denmark

**DOI:** 10.1186/s12866-015-0589-z

**Published:** 2015-10-30

**Authors:** Chad A. Keyser, Henrik H. De Fine Licht, Bernhardt M. Steinwender, Nicolai V. Meyling

**Affiliations:** Department of Plant and Environmental Sciences, University of Copenhagen, Thorvaldsensvej 40, 1871 Frederiksberg, Denmark

**Keywords:** AFLP, Entomopathogenic fungi, Soil baiting, Rhizosphere competence, Population ecology

## Abstract

**Background:**

Knowledge of the natural occurrence and community structure of entomopathogenic fungi is important to understand their ecological role. Species of the genus *Metarhizium* are widespread in soils and have recently been reported to associate with plant roots, but the species *M. flavoviride* has so far received little attention and intra-specific diversity among isolate collections has never been assessed. In the present study *M. flavoviride* was found to be abundant among *Metarhizium* spp. isolates obtained from roots and root-associated soil of winter wheat, winter oilseed rape and neighboring uncultivated pastures at three geographically separated locations in Denmark. The objective was therefore to evaluate molecular diversity and resolve the potential population structure of *M. flavoviride*.

**Results:**

Of the 132 *Metarhizium* isolates obtained, morphological data and DNA sequencing revealed that 118 belonged to *M. flavoviride*, 13 to *M. brunneum* and one to *M. majus*. Further characterization of intraspecific variability within *M. flavoviride* was done by using amplified fragment length polymorphisms (AFLP) to evaluate diversity and potential crop and/or locality associations. A high level of diversity among the *M. flavoviride* isolates was observed, indicating that the isolates were not of the same clonal origin, and that certain haplotypes were shared with *M. flavoviride* isolates from other countries. However, no population structure in the form of significant haplotype groupings or habitat associations could be determined among the 118 analyzed *M. flavoviride* isolates.

**Conclusions:**

This study represents the first in-depth analysis of the molecular diversity within a large isolate collection of the species *M. flavoviride*. The AFLP analysis confirmed a high level of intra-specific diversity within the species and lack of apparent association patterns with crop or location indicates limited ecological specialization. The relatively infrequent isolation of *M. flavoviride* directly from crop roots suggests low dependence of root association for the species.

**Electronic supplementary material:**

The online version of this article (doi:10.1186/s12866-015-0589-z) contains supplementary material, which is available to authorized users.

## Background

The predominantly entomopathogenic fungal genus *Metarhizium* (Hypocreales: Clavicipitaceae) has a global distribution [1–3] and several species have been intensely researched for their pest control potential. Discordantly, limited focus has been given to the assessment of natural occurrence and distribution of this widespread fungal genus. Knowledge of the compositions of entomopathogenic fungal communities and structure of their populations is important to understand their ecological function and contribution to host regulation and potential for conservation biological control [4].

Evaluation of fungal diversity and community structure depends heavily on criteria for species identification. With few morphological distinct features, species referred to as *M. anisopliae* and variants thereof in the literature could belong to several different species if no explicit molecularly based identification has been conducted. A revised taxonomy of the *M. anisopliae* lineage based on a multigene phylogeny resolved nine species within what could be considered *M. anisopliae sensu lato* if based solely on morphology [5]. Recently, the taxonomy of the whole genus *Metarhizium* was revised leading to the inclusion of other genera as well as elevation of some of the former *M. flavoviride* variants to species level [1]. Kepler and Rehner [6] further specified additional genomic regions for species identification. Molecularly based evaluation of the structure of *Metarhizium* communities should therefore be included in studies of natural occurrence and distribution to provide full recognition of diversity.

In temperate climatic regions, *Metarhizium* spp. are predominantly isolated from the soil environment [7]. Surveys of entomopathogenic fungi from soil samples conducted at different geographical locations have shown that *M. anisopliae s.l.* can be abundantly isolated from managed ecosystems [8–15]. However, few studies have evaluated *Metarhizium* community structure using explicit molecular characterization. In Denmark, Meyling et al. [13] isolated *M. anisopliae s.l.* from an agricultural field and Steinwender et al. [14] subsequently showed that the *Metarhizium* community was composed of several species, predominantly *M. brunneum* followed by *M. robertsii*.

In the soil environment, naturally occurring *Metarhizium* spp. may also be isolated from roots of different plants [16, 17]. Some level of plant association was indicated by a predominance of *M. robertsii* found on roots of herbaceous plants as compared to *M. brunneum,* which was mostly recovered from roots of woody plants [17]. Sampling roots of different plants from an ecosystem might therefore inform about the *Metarhizium* diversity of an area and reveal potential habitat associations related to plants growing at the site. It was recently shown that similar *M. brunneum* and *M. robertsii* multilocus genotypes can be repeatedly isolated from roots and soil samples at the same site [18]. However, the structure of the entomopathogenic fungal community in the soil can differ markedly between agricultural fields within relatively close proximity and characterization of a single site may not provide a general distribution pattern. For example, Meyling et al. [13] reported of high frequency of occurrence of *M. anisopliae s.l.* (predominantly *M. brunneum*) at a site in Denmark, while another Danish agricultural field revealed a low occurrence of *M. anisopliae s.l.* [19] using same isolation methods. At the latter site, *M. flavoviride* was isolated more frequently than *M. anisopliae s.l.* indicating location specific entomopathogenic fungal communities.

The aim of the current study was to examine the natural occurrence and diversity of *Metarhizium* spp. isolates obtained from roots and root associated soil of different common crops at geographically separated agricultural fields in Denmark. Fields with oil seed rape, winter wheat as well as permanent grassland were each sampled from three distinct geographical locations. Using such selection criteria, we expected to reveal potential crop and/or location associations of the *Metarhizium* populations from these three distinct geographical sites. The study resulted in a predominance of *M. flavoviride* isolates which was contrary to our expectations to isolate *M. brunneum* most frequently based on recent evidence [14]. *Metarhizium flavoviride* has not previously been studied for within-species diversity, thus the intraspecific variability was examined using Amplified Fragment Length Polymorphism (AFLP) markers to evaluate diversity and potential habitat and/or geographical associations.

## Results

### *Metarhizium* distribution and species diversity

A total of 132 *Metarhizium* isolates were acquired from the soil samples collected at nine agricultural fields (Table 1). Fourteen of the isolations were obtained using selective media; the remaining 118 isolates were attained using standard soil baiting with *Tenebrio molitor* larvae. The overall distribution of isolates by area and crop type was not homogenous (*χ*^2^ = 56.8770; df = 4; *P* < 0.0001). A total of 34 isolates were from the Fårevejle area, 71 isolates from Skibby and 27 isolates from Tåstrup; the resulting frequency distribution by area was significantly heterogeneous (*χ*^2^ = 35.9563; df = 2; *P* < 0.0001). Similarly, significantly different frequency distributions were found when compared by crop type (*χ*^2^ = 12.8636; df = 2; *P* = 0.0016).

**Table 1:** Total number of *Metarhizium* spp. isolations from soil samples collected at three locations in Denmark

Location	Crop	*M. brunneum*	*M. flavoviride*	*M. majus*	Total *Metarhizium* spp. isolated
Fårevejle					
	Oilseed rape	0	17	0	17
	Winter wheat	0	17 (1)	0	17
	Grass pasture	0	0	0	0
Skibby					
	Oilseed rape	1	5	0	6
	Winter wheat	2	19 (1)	0	21
	Grass pasture	6 (5)	37 (5)	1 (1)	44
Tåstrup					
	Oilseed rape	1	1	0	2
	Winter wheat	3	16 (1)	0	19
	Grass pasture	0	6	0	6
Total isolates		13 (5)	118 (8)	1 (1)	132 (14)

Each location is represented by three crop types. Three different species were identified and isolates for each are presented and summed. The numbers represent isolates obtained both by insect baiting using *Tenebrio molitor* larvae and by plating of root homogenate on selective agar media (numbers in brackets represent the isolates obtained on selective media)

**Fig. 1:**
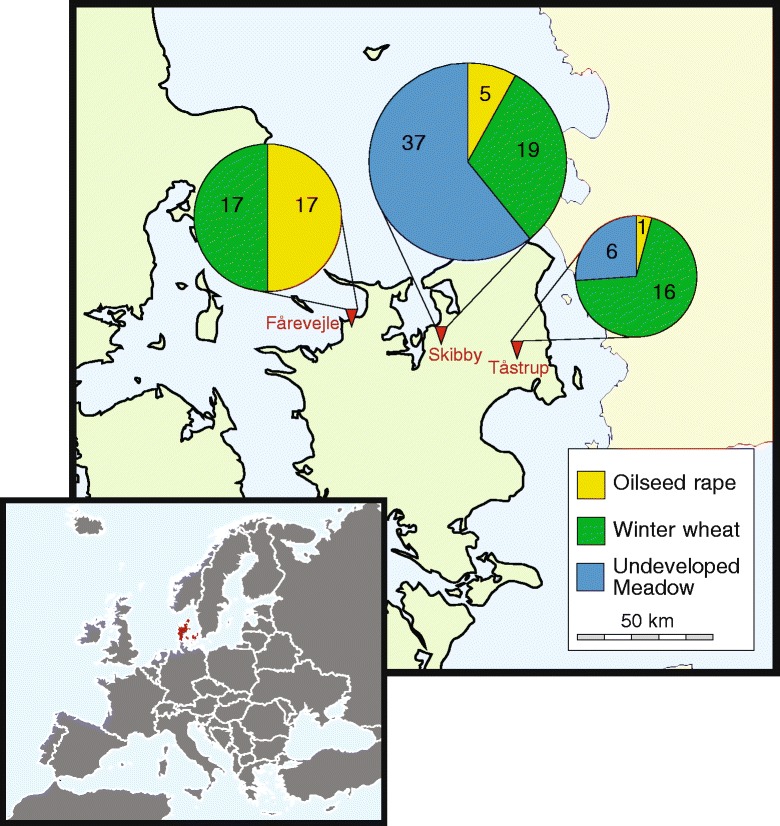
Map depicting geographical location of the three sampled localities at the island of Zealand, Denmark. Map of Europe is inserted. At each locality, three fields were sampled for 50 root systems and associated soil of each of the respective crops, i.e. oilseed rape, winter wheat and uncultivated pasture. Inserted pie charts indicate number of *Metarhizium flavoviride* isolates obtained from each field. Areas of pie charts indicate relative contribution of each locality to the total number of isolates characterized. Maps were created from free maps available at d-maps.com (http://d-maps.com/carte.php?num_car=2233&lang=en and http://d-maps.com/carte.php?num_car=5116&lang=en)

Of the 132 isolates, 118 were morphologically identified as *M. flavoviride* based on their characteristic bright green colony color and conidial dimensions. The remaining 14 isolates were morphologically classified as belonging to *M. anisopliae s.l.* The *M. flavoviride* isolates were collected from all fields except the uncultivated pasture in Fårevejle (Fig. 1), which had more moist soil than at the other areas.

**Fig. 2:**
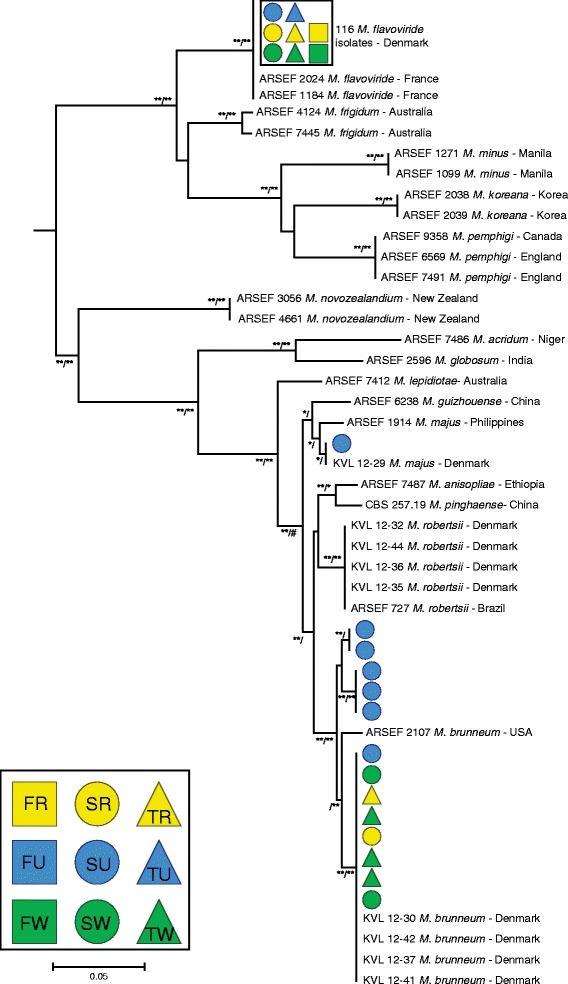
Phylogenetic tree of *Metarhizium* spp. based on sequence alignment of the nuclear intergenic region MzFG543igs. Tree based on Bayesian analysis is shown with branch support for both Bayesian and Maximum-likelihood analyses; Bayesian (100 = **, 95 < *) / Maximum-likelihood (100 = **, 95 < *; # denotes where branching topology did not agree between Bayesian and Maximum-likelihood analysis). 130 *Metarhizium* spp. isolates representing three species were sequences for MzFG543igs. Colored symbols indicate location and crop from which the isolate was acquired [F = Fårevejle (*square*), S = Skibby (*circle*), T = Tåstrup (*triangle*), R = oilseed rape (*yellow*), U = un-cultivation pasture (*blue*), and W = winter wheat (*green*)]

To verify species identity, 130 of the isolates collected were sequenced for the intergenic region MzFG543igs located between an ATP synthase gamma chain and NAD binding protein encoding genes and phylogenetically analyzed. Species branches of the resulting phylogenetic tree (Fig. 2) were supported by both Bayesian and Maximum-likelihood analyses with 13 isolates clustering with *M. brunneum*. The *M. brunneum* isolates were distributed among three separate clades with two clades represented by isolates from the undeveloped field at Skibby, while eight isolates representing all the three geographical areas clustered with a clade previously found in Denmark (Fig. 2). The 116 *M. flavoviride* isolates that were sequenced for MzFG543igs all shared the same sequence which was similar to isolates from France (Fig. 2). The single isolate of *M. majus* originated from plating of root homogenate on selective agar medium collected at the undeveloped field at Skibby.

### Intraspecific variation of *M. flavoviride*

**Fig. 3:**
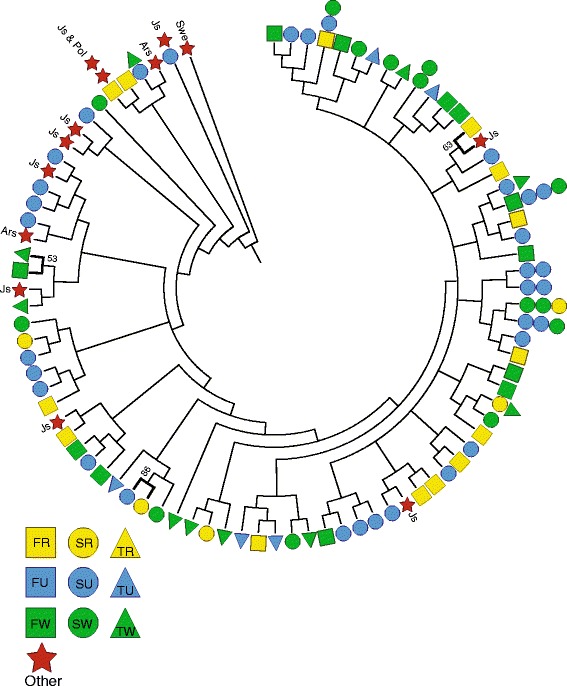
Dendrogram based on 30 AFLP loci from 106 *Metarhizium flavoviride* isolates. The dendrogram was generated with the Phylip Package using Dice coefficient with 1000 bootstrap replicates. Bolded branches represent significant pairwise clusters based on bootstrapping. Colored symbols indicate location and crop from which the isolate was acquired [F = Fårevejle (*square*), S = Skibby (*circle*), T = Tåstrup (*triangle*), R = oilseed rape (*yellow*), U = un-cultivation pasture (*blue*), W = winter wheat (*green*). Isolates originating from other locations not sampled in the present study included (red star): Js = Jutland, Denmark; Swe = southern Sweden; Ars = Årslev; and Pol = Poland]

Amplified fragment length polymorphism (AFLP) analysis was performed using 93 of the *M. flavoviride* isolates collected in this study as well as 13 isolates collected from different geographical locations, including: Jutland, Denmark (Js: *n* = 9), Årslev, Denmark (Ars: *n* = 2) [14], Poland (Pol: *n* = 1), and southern Sweden (Swe: *n* = 1). The AFLP analysis produced 230 polymorphic loci; however, based on the scoring criteria and inconsistency between dual replicates, only 30 repeatable and consistent loci were selected for inclusion in the analysis. Using the unweighted pair group method with arithmetic mean (UPGMA) analysis, intraspecific variation among the *M. flavoviride* isolates was quite high identifying 78 haplotypes among the 93 analyzed isolates, of which 11 were shared among 2–4 isolates from different areas while the remaining were unique (Fig. 3). However, only three significant AFLP haplotype clusters could be identified by bootstrapping and thus area or crop associated population structure could not be established.

## Discussion

The majority of *Metarhizium* related research has favored species of the *M. anisopliae* complex; in contrast *M. flavoviride* are often reported as being infrequently isolated [8–10, 12, 16, 17, 20, 21] and this species has not been studied in detail. Meyling and Eilenberg [19] have reported significant occurrence of *M. flavoviride* in Denmark within an agroecosystem located close to the fields sampled in Tåstrup in the present study. However, studies at another location in Denmark revealed a predominance of *M. brunneum* isolates using similar isolation methods [7, 14]. In the present study we surveyed three geographically separate areas and found *M. flavoviride* to be most abundant at all three areas, although at different frequencies of occurrence, indicating that the relatively high frequency of *M. flavoviride* reported by Meyling and Eilenberg [19] was unlikely to be a one-off event. Clearly the species composition of *Metarhizium* communities is not ubiquitous or random at the studied geographical scales, and further in-depth ecological studies are necessary to identify the driving forces that determine *Metarhizium* spp. distribution in nature.

The predominance of *M. flavoviride* observed in the present study was unexpected; our initial intention was to analyze the within-species diversity using microsatellites or simple sequence repeat (SSR) markers as done previously [14]; these markers have been shown to be highly suitable for explicit multilocus genotyping [22]. However, the SSR markers developed for *M. anisopliae s.l.* were observed to have a high frequency (>40 %) of null alleles when tested on *M. flavoviride* isolates and no polymorphism [14, 18], and were therefore unsuitable for diversity evaluation within this species. Thus, to characterize the intraspecific variability within *M. flavoviride* and evaluate the diversity we chose to use AFLP markers. Diversity assessment using AFLP markers has been used previously for other *Metarhizium* spp. isolate collections [10, 23]. Inglis et al. [10] used AFLP markers to determine the diversity within *M. anisopliae s.l.* from western Canada, and they were able to identify several distinct haplotypes. It is important to note however that this was done prior to the Bischoff et al. [5] taxonomic revision and the material evaluated by Inglis et al. [10] has later been split into separate species, predominantly *M. brunneum* (T. Kabaluk, pers. comm.). Fernandes et al. [23] used AFLP markers to evaluate inter- and intra-species variability among isolates of several *Metarhizium* species; they found that the method revealed high level of diversity but only clear clustering within *M. acridum* was seen. Reliable species identification placed within an authoritative molecular phylogeny is important when applying general markers such as AFLP to evaluate intra-specific diversity of *Metarhizium* spp.

In contrast to the relatively few *M. brunneum* isolates sequenced in the current study, all *M. flavoviride* isolates shared the same sequence at the selected genomic intergenic region, even among spatially distant sampled isolates. This indicates that *M. flavoviride* may not be composed of phylogenetically distinct clades as is evident for *M. brunneum* [14] and *M. robertsii* [15]. In the present data set *M. brunneum* was also found to be represented by three distinct clades, all co-occurring at the Skibby site. AFLP characterizations of the *M. flavoviride* isolates included only repeatedly reproduced markers since reproducibility is known to be a challenge with amplified fragments from restriction enzyme markers [22]. The relatively low number of loci in the AFLP analysis is likely to have resulted in the limited statistical support for the haplotype groupings. However, the UPGMA analysis clearly revealed diversity within the species indicating that the isolates are not of the same immediate clonal origin, but also that *M. flavoviride* isolates from other countries shared haplotypes with some of the isolates of the present study. This could tentatively indicate that all the analyzed samples originated from a single diverse population of clonal lineages with little local adaptation. The lack of host specialization and the ability to subsist as a pathogen or saprophyte may result in a reduced rate of adaption, due to limited selection pressure in particular habitats and the advantage of maintaining broad host and habitat ranges. Future development of species-specific markers (i.e., microsatellite SSR markers) would however improve the ability to evaluate *M. flavoviride* diversity and determine more evidently any potential habitat associations.

In line with other studies, a single species of *Metarhizium* was found to be the most prevalent in a given area [10, 14, 16, 17], however it is unclear what factors contribute to this high frequency in the community. *Metarhizium* spp. are able to acquire nutrients as an insect pathogen or as a plant root associate, which makes it reasonable to suggest that an aptitude at either life strategy would favor a particular species. It has been observed that an isolate of *M. flavoviride* (KVL 14–112) had lower virulence than *M. brunneum* or *M. robertsii* isolates towards *T. molitor* larvae [24] suggesting that *M. flavoviride* may possess relatively low virulence towards at least some insects. However, Meyling et al. [13] reported of relatively frequent natural mycosis by *M. flavoviride* in aboveground weevils suggesting that the fungus plays a role in the regulation of some insect populations. At the site investigated by Meyling et al. [13] *M. brunneum* was most frequently isolated by insect baiting of soil samples, but absent as mycosis in aboveground hosts. We implemented two standard methods for the isolation of entomopathogenic fungi in this study, viz., a soil bait method with *T. molitor* and an in vitro selective media method. Of the two, the soil bait method was most effective in recovering isolates of *M. flavoviride*. The selective media method used was also employed by Wyrebek et al. [17] to isolate *Metarhizium* spp. from roots of different plants. Although Wyrebek et al. [17] did not report of the isolation of *M. flavoviride* from roots, Behie and Bidochka [25] reported that *M. flavoviride* was able to associate with plant roots in the rhizosphere and transfer nitrogen to the plant from insect cadavers. However, in the present study we sequenced the same isolate (ARSEF 9358) used by Behie and Bidochka [25] which appeared to be *M. pemphigi* (previously *M. flavovoride* var. *pemphigi*), and it thus remains undetermined if *M. flavoviride* can associate with plant roots. The reduced number of isolates of *M. flavoviride* collected on selective media from roots in the present study might indicate that, while present in the soil environment, *M. flavoviride* was not associating closely with the roots of the crop plants, but neither were other *Metarhizium* species. Further studies are needed to confirm these observations and investigate what might be driving the *M. flavoviride* predominance at the investigated field sites. However, the current data and that of Meyling et al. [13] indicate that entomopathogenicity is an important ecological trait for *M. flavoviride* occurrence and distribution in agroecosystems.

## Conclusion

The present study revealed that *M. flavoviride* was the predominant *Metarhizium* species in the soil environment at all three agricultural localities investigated in Denmark each represented by three fields, and that the fungal isolates were mainly obtained using *T. molitor* baiting. AFLP analysis of the *M. flavoviride* isolates revealed a high level of intra-specific diversity within *M. flavoviride*, with some widespread AFLP haplotypes also occurring in other countries. This is the first report of an in-depth analysis of the molecular diversity within a large isolate collection of the entomopathogenic fungal species *M. flavoviride* which could lay the foundations for new research of this little investigated fungus.

## Methods

### Field sampling

Three geographically separated areas were located on the island of Zealand, Denmark (Fig. 1), including: Fårevejle (55.78° N, 11.43 °E), Skibby (55.75 ° N, 11.99 °E) and Tåstrup (55.67 ° N, 12.3 °E). Within each area three agricultural fields were identified resulting in a 2-level nested design. Each site included one field of winter wheat (*Triticum aestivum*; var. JB Asano at Fårevejle, var. Jensen at Skibby, var. Tabasco at Tåstrup), one with winter oilseed rape (*Brassica napus* var. DK-Expower at all sites), and a permanent grass pasture that had been without cultivation for more than 20 years.

From each of the nine fields, 50 root samples were collected by walking an approximately 200 m transect from the edge of the field towards the center; a combined root/soil sample was collected every 3–4 m using a long-handled weeding fork. Each sample was placed individually in a pre-labeled 10 l polyethylene bag along with 300–500 g of root-associated soil adhering to the root system. The three fields from each geographic area were sampled on the same day; collections were made on three consecutive days from July 23 to July 25, 2013. After collection samples were placed at 5 °C until they were processed within 30 days.

### Fungal isolation

*Selective media*: From each sample a section of the root was removed and washed with ddH_2_O to remove loose soil. A portion (~0.5 ± 0.25 g) of the washed root was then cut in small pieces (~2 mm) and placed in 10 ml ddH_2_O and homogenized using a rotary homogenizer [17]. 200 μl of homogenate was then spread onto a selective agar media adapted from Fernandes et al. [26], which consisted of: 39 g potato dextrose agar (Sigma Chemical, MO, USA), 1 g yeast extract (Merck KgaA, Darmstadt Germany), 0.25 g cyclohexamide (Sigma Chemical, MO, USA), 0.5 g chloramphenicol (Sigma Chemical, MO, USA), 0.02 g dodine (Agriphar, Belgium) (all weights per liter); two plates for each root sample were prepared. After inoculation the plates were incubated for 28 days at 22 °C in darkness and checked on day 7, 14, 21 and 28. Colonies morphologically identified as *Metarhizium* were transferred to a clean plate of potato dextrose agar supplemented with 1 % yeast extract (PDAY) using a sterile inoculating needle to obtain pure cultures.

*Soil baiting*: The soil baiting method used in this study was adapted from Zimmermann [27]. The soil from each sampling site was allowed to air dry over night at room temperature and then re-moistened with ddH_2_O so that it was “slightly damp”. Soil (~120 ml) was then placed in a plastic cup (155 ml) leaving 1 cm of airspace at the top. Prior to filling the cup large debris and clumps were removed or broken up. Ten healthy 4–5th instar *Tenebrio molitor* L. (Coleoptera: Tenebrionidae) larvae were then added to each cup. A lid with ventilation holes was placed on top of each sample and the cups were stacked upside down – so that the larvae were beneath the soil – in a box. Every 1–2 days the box was rotated so that the orientation of the cups shifted, forcing the *T. molitor* larvae to move through the soil substrate increasing the likelihood they would come in contact with entomopathogens in the soil. Insect survival was checked weekly; dead insects were removed, washed with ddH_2_O and placed in a medicine cup with a moist piece of filter paper to check for mycosis. Using a sterile inoculation needle, fungal isolations were made from insect cadavers with mycosis and plated on PDAY media. After 14 days growth colonies were evaluated, morphologically identified and *Metarhizium* spp. isolates were kept for further studies.

The frequency distributions of *Metarhizium* isolates were tested for homogeneity among sampling area and crop type using Pearson’s *χ*^2^ tests in R performed in RStudio (version 0.97.551).

### PCR amplification and sequencing

For each *Metarhizium* isolate, lyophilized mycelium was prepared by growing cultured isolates in liquid media [14] for four days at 22 °C on a stirring table at 170 rpm. Mycelium was separated using a vacuum filtration system, frozen over night at −20 °C and lyophilized. DNA was extracted from the lyophilized mycelia using the DNeasy Plant Mini Kit (QIAGEN, Hilden, Germany) following the manufacturer’s instructions.

The PCR amplification and sequencing methods in this study were of the intergenic region MzFG543igs region flanked by the coding regions of the genes ATP synthase gamma chain and NAD binding protein according to Kepler and Rehner [6]. The master mix reagents for each PCR reaction consisted of: 32 μl milliQ H_2_O, 5 μl Taq DNA buffer, 1 μl dNTP, 1 μl Taq DNA polymerase, 5 μl of each primer, and 1 μl DNA sample. The primers used were MzFG543igs_1F (5′-ATT CAT TCA GAA CGC CTC CAA-3′) and MzFG543igs_4R (5′-GGT TGC GAC TCA CAA TCC ATG-3′). PCR amplification was initiated by denaturation at 95 °C for 2 min followed by 40 cycles of three steps including: 95 °C denaturation for 30 s, 62 °C annealing for 30 s, and 72 °C extension for 60 s; after the 40 cycles the final step was 72 °C extension for 15 min. The PCR products were visualized on 1.5 % agarose gel to ensure strong single bands and purified using the Illustra™ GFX™ PCR DNA and Gel Band Purification Kit. The purified PCR products were sent to Beckman Coulter Genomics (UK) for sequencing with the PCR primers. The sequence chromatograms were manually corrected and aligned using ClustalW [28] with default settings as implemented in BioEdit 7.1.3 [29]. Additionally, sequences of known *Metarhizium* spp. isolates from Kepler et al. [1] in addition to three *Metarhizium* spp. isolates from the ARSEF collection (i.e., ARSEF nos. 1184, 1271 and 9358) were included in the phylogenetic analyses. Sequences are deposited in GenBank (Table 2). The ~900 base pair multiple alignment was analyzed using ModelTest to determine the optimal DNA substitution model and evaluated with AIC scores as implemented in Topali [30]. The phylogeny was inferred with maximum likelihood estimation using RaxML with 500 bootstrap replicates and Bayesian analyses using MrBayes ver3.1 and executed from within Topali. The final dendrogram was depicted suing MEGA5 [31].

**Table 2:** List of selected *Metarhizium* spp. isolates from Denmark characterized in the present study

Species	Isolate accession	Isolate origin	GenBank accession	References
*Metarhizium flavoviride*	KVL 14–112	Undeveloped pasture, *T. molitor* bait larva, Skibby	KT335973	This study; [24]
*M. majus*	KVL 14–21	Undeveloped pasture, SM, Skibby	KT335974	This study
*M. robertsii*	KVL 12–32	Organic field, *T. molitor* bait larva, Årslev	KT335975	This study; [14]
*M. brunneum*	KVL 14–08	Undeveloped pasture, SM, Skibby	KT335976	This study
*M. brunneum*	KVL 14–10	Undeveloped pasture, SM, Skibby	KT335977	This study
*M. brunneum*	KVL 14–40	Wheat, *T. molitor* bait larva, Skibby	KT335978	This study

Isolates were obtained by in vitro culturing on selective agar media (SM) or by bating soil samples with mealworm larvae (*T. molitor*)

### Intraspecific variation of *M. flavoviride*

New DNA extractions with a greater quantity of DNA were made from the lyophilized mycelium. This was accomplished using the Qiagen DNeasy Plant Mini Kit with the following protocol modifications: A small amount of lyophilized material (~2-3 “match-head” size pieces) was placed in a 2 ml Eppendorf vial, which had been prepared with 1.0 mm and 2.5 mm dia. zirconia-glass beads (Biospec Products). Next, 600 μl buffer AP1 from the Qiagen DNeasy Plant Mini Kit (cat. no. 69104) was added and the samples homogenized in a FastPrep machine (BIO101/Savant FP120 FastPrep, Qbiogene, CA, USA) and shaken twice for 35 s at 5.0 speed. The samples were then removed and 6 μl of proteinase K and 6 μl RNase A from the Qiagen DNeasy Plant Mini Kit were added and incubated at 65 °C for 1 h during which they were manually shaken every 15 min. After incubation, 195 μl buffer P3 was added and they were incubated on ice for 5 min. The remaining steps were according to the protocol and the DNA was eluted in 50 μl buffer AE. The quantity and quality of DNA available in the extracted material was quantified using a NanoDrop 1000 (Thermo Scientific).

The Amplified Fragment Length Polymorphism (AFLP) procedures were performed according to Vos et al. [32], and the amount of DNA per reaction were standardized to 100 ± 20 ng. Initially the DNA was digested by two restriction enzymes (EcoRI and MseI) and oligonucleotide adapters attached to the resulting ends. This was accomplished by preparing two master mixes, master mix A contained: 0.1 μl MseI (10 u/μl), 0.25 μl Eco RI (20 u/μl), 0.1 μl T4 ligase (6 Weiss u/μl), 0.1 μl T4 ligase buffer, 0.05 μl diluted (1 mg/ml) BSA, 0.1 μl 0.5 M NaCl, and 0.3 μl milliQ H_2_O, for each reaction. Master mix B contained: 1 μl 10× T4 ligase buffer, 1 μl 0.5 M NaCl, 0.5 μl diluted BSA (1 mg/ml), 1 μl MseI adaptor pair, 1 μl EcoRI adaptor pair, for each reaction. Prior to preparing master mix B, the MseI and EcoRI adaptor pairs were heated to 95 °C for 5 min and then allowed to cool at room temperature for 10 min; the MseI adaptor pair consisted of a 1:1 combination of M-ADAP I (5′-GAC GAT GAG TCC TGA G-′3) and M-ADAP II (5′-TAC TCA GGA CTC AT-′3) and the EcoRI adaptor pair consisted of a 1:1 combination of E-ADAP I (5′-CTC GTA GAC TGC GTA CC-′3) and E-ADAP II (3′-CAT CTG ACG CAT GGT TAA-′5) at a concentration of 20 μM each (so after combination the concentration of each is 10 μM). After preparation master mix B was added to master mix A and mixed before 5.5 μl of DNA was added so that the total volume was 11 μl. This was then incubated at 37 °C for 2 h, after which 189 μl of milliQ H_2_O was added.

A PCR preamplification of the restriction-ligation products consisted of: 12.55 μl milliQ H_2_O, 4 μl Phusion buffer (Finnzymes), 0.4 μl dNTP (10 μM), 0.25 μl Phusion polymerase (Finnzymes), 0.4 μl Eco pre-amp primer (5′-GAC TGC GTA CCA ATT CA-′3), and 0.4 μl Mse pre-amp primer (5′-GAT GAG TCC TGA GTA AC-′3). To this mixture 2 μl of the product from the cut ligation above was added. The DNA fragments were then amplified using the following PCR program: 98 °C denaturation for 30 s, followed by 35 cycles next three steps, 98 °C denaturation for 30 s, 56 °C annealing for 30 s, and 72 °C extension for 1 min; after the 35 cycles the final step was 72 °C extension for 120 s. A 1:10 dilution of the preamp product was then made and used in the selective amplification step.

A selective amplification mixture was prepared which consisted of: 11.85 μl milliQ H_2_O, 4 μl Phusion buffer, 0.4 μl dNTP (10 μM), 0.25 μl Phusion polymerase, 1 μl Eco sel-amp primer with FAM labeled (10 μM) (5′-GAC TGC GTA CCA ATT CAC C-′3), 0.5 μl Mse sel-amp CAC primer (20 μM) (5′-GAT GAG TCC TGA GTA ACA C-′3), and 0.5 μl Mse sel-amp CAT primer (20 μM) (5′-GAT GAG TCC TGA GTA ACA T-′3). To this 1.5 μl of the diluted pre-amplification product was added to bring the total volume to 20 μl. The following PCR program was performed: for 8 cycles, 98 °C denaturation for 30 s, 65 °C annealing for 30 s, and 72 °C extension for 90 s, reducing the annealing temperature 1 °C each cycle; then 24 cycles of: 98 °C denaturation for 30 s, 56 °C annealing for 30 s, and 72 °C extension for 90 s, followed by 72 °C for 7 min. This was then visualized on 1.5 % agarose gel to insure the presence of a distinct smear and banding pattern.

The selective amplification products were prepared for fragment length analysis by mixing: 0.1 μl sample in 8.7 μl formamide and 0.3 μl Genescan Rox500 (Applied Biosystems). The samples were then analyzed on ABI Prism® 3100 Genetic Analyzer (Applied Biosystems, Foster City, CA). The entire AFLP procedure was performed twice using the same extracted DNA resulting in two technical replicates for each isolate. Because the samples were run on more than one 96-well plate, a specific isolate was included on all plates to control for plate-specific variation. Presence of bands were scored as follows using GeneMapper® software 5 (Applied Biosystems) and ROX500 as internal size standard: AFLP fragments were scored as present (1) if they had a signal intensity greater than 100 in duplicate samples, or if there was a disagreement between the two replicates and one was above 100 and the other was above 50. In contrast, if an AFLP fragment had signal intensity less than 100 for both isolate replicates or if one was below 50 then it was considered absent (0). A signal intensity of 50 was considered the absolute minimum to be reliably scored and distinguished from background noise. A cluster analysis of the resulting binomial data set was performed by calculating the pairwise distance measure using Dice coefficient with 1000 bootstrap replicates using DistAFLP (available at: http://pbil.univ-lyon1.fr/ADE-4/microb/) [33]. These bootstrap trees were then analyzed using the Neighbor and Consensus executables in the Phylip Package [34] to produce a tree with bootstrap support based on the unrooted unweighted pair group method with arithmetic mean (UPGMA) [35].

## Availability of supporting data

Representative DNA sequences obtained in the present study were deposited in GenBank, National Center for Biotechnology Information (NCBI) under accession numbers KT335973-KT335978 (http://www.ncbi.nlm.nih.gov/). The AFLP scoring matrix used for the UPGMA analysis in Fig. 3 is available as Additional file 1.

## Additional file

Additional file 1:
**Fragment scoring matrix (presence = 1, light green; absence = 0) of Amplified Fragment Length Polymorphism (AFLP) data.** Only fragments positive in duplicate runs were scored as present. Results of analysis of AFLP data are presented in Fig. 3. (XLSX 21 kb)

## Abbreviations

AFLP: amplified fragment length polymorphism
AIC: akaike information criterion
ARSEF: The USDA-ARS collection of entomopathogenic fungal cultures
NAD: nicotinamide adenine dinucleotide
PDAY: potato dextrose agar supplemented with 1 % yeast extract
UPGMA: unweighted pair group method with arithmetic mean
SSR: simple sequence repeats
